# 516. Cytomegalovirus (CMV) Saliva Shedding Kinetics in Children with Congenital Cytomegalovirus Infection (cCMV)

**DOI:** 10.1093/ofid/ofae631.168

**Published:** 2025-01-29

**Authors:** Swetha Pinninti, Sunil Pati, Zdenek Novak, Karen Fowler, Suresh Boppana, Shannon Ross

**Affiliations:** Heersink School of Medicine/University of Alabama at Birmingham, Birmingham, Alabama; University of Alabama at Birmingham, Birmingham, Alabama; University of Alabama at Birmingham, Birmingham, Alabama; University of Alabama at Birmingham, Birmingham, Alabama; University of Alabama at Birmingham, Birmingham, Alabama

## Abstract

**Background:**

CMV is the most common cause of congenital viral infection worldwide and the leading cause of non-genetic sensorineural hearing loss (SNHL). Children with congenital CMV infection (cCMV) shed the virus in urine and saliva for prolonged but variable durations. However, data on CMV saliva shedding kinetics in cCMV is limited because of the lack of longitudinal virological follow-up. The objective of this study is to describe CMV shedding kinetics in saliva during early childhood in a cohort of children with cCMV identified through universal newborn screening.

**Figure:**
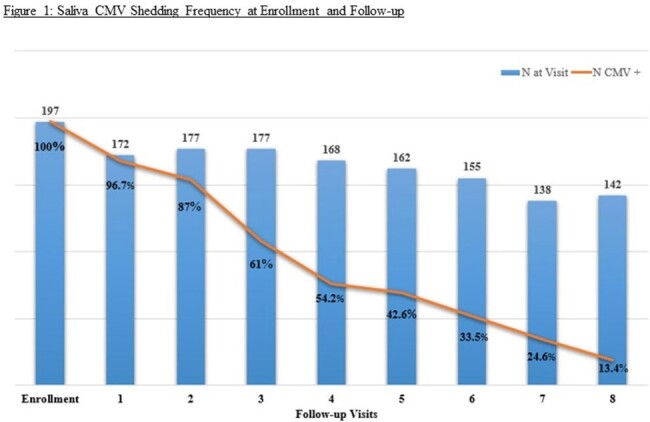

**Methods:**

As part of the CMV and Hearing Multicenter Screening study (CHIMES), >100,000 newborns were screened for cCMV. Data from 211 children with ≥ 5 visits each with audiological follow-up and serial saliva samples collected every six months through four years of life was analyzed. Saliva CMV shedding duration and intermittent shedding according to presence of newborn symptoms, hearing status and antiviral treatment are described.

**Results:**

Of the 211 children with cCMV, 14 received anti-viral treatment in early infancy. Most children shed CMV in saliva during infancy with a gradual decrease in frequency on follow-up (Figure 1). The median duration of shedding did not differ between children with asymptomatic and symptomatic cCMV (25 vs 26 months respectively; p = 0.61); children with SNHL and normal hearing (22 vs 26 months respectively; p = 0.26); and between those treated with antiviral agents and untreated children (23 vs 25 months; p = 0.9). Salivary CMV shedding was intermittent in about a third of the cohort (71/197 - 36%), mostly (91.5%) in those with asymptomatic cCMV. CMV shedding duration was significantly longer in children with intermittent shedding compared to those who did not (34 vs 17 months; p < 0.0001).

**Conclusion:**

In this large cohort of children with cCMV identified through newborn screening, children shed CMV DNA in saliva for a median of two years, irrespective of newborn symptoms, hearing status, and receipt of anti-viral therapy. A third of the cohort shed CMV intermittently. Based on these observations, we conclude that a negative saliva CMV PCR result during infancy in newborns with findings consistent with cCMV but not tested in the newborn period likely excludes a diagnosis of cCMV.

**Disclosures:**

**Swetha Pinninti, MD**, Moderna: Grant/Research Support|Pfizer: Grant/Research Support **Karen Fowler, DrPH**, Moderna: Advisor/Consultant **Suresh Boppana, MD**, GSK: Advisor/Consultant|Merck: Grant/Research Support|Pfizer: Grant/Research Support

